# The Clinical Relevance of Serum NDKA, NMDA, PARK7, and UFDP Levels with Phlegm-Heat Syndrome and Treatment Efficacy Evaluation of Traditional Chinese Medicine in Acute Ischemic Stroke

**DOI:** 10.1155/2015/270498

**Published:** 2015-10-11

**Authors:** Xiuxiu Han, Yonghong Gao, Bin Ma, Ying Gao, Yikun Sun, Ru Jiang, Yayun Wang

**Affiliations:** ^1^Department of Neurology, Dongzhimen Hospital Affiliated to Beijing University of Chinese Medicine, Beijing 100700, China; ^2^Key Laboratory of Chinese Internal Medicine of Ministry of Education, Dongzhimen Hospital Affiliated to Beijing University of Chinese Medicine, Beijing 100700, China

## Abstract

According to the methods of Patient-Reported Outcome (PRO) based on the patient reports internationally and referring to U.S. Food and Drug Administration (FDA) guide, some scholars developed this PRO of stroke which is consistent with China's national conditions, and using it the feel of stroke patients was introduced into the clinical efficacy evaluation system of stoke. “Ischemic Stroke TCM Syndrome Factor Diagnostic Scale (ISTSFDS)” and “Ischemic Stroke TCM Syndrome Factor Evaluation Scale (ISTSFES)” were by “Major State Basic Research Development Program of China (973 Program) (number 2003CB517102).” ISTSFDS can help to classify and diagnose the CM syndrome reasonably and objectively with application of syndrome factors. Six syndrome factors, internal-wind syndrome, internal-fire syndrome, phlegm-dampness syndrome, blood-stasis syndrome, qi-deficiency syndrome, and yin-deficiency syndrome, were included in ISTSFDS and ISTSFES. TCM syndrome factor was considered to be present if the score was greater than or equal to 10 according to ISTSFDS. In our study, patients with phlegm-heat syndrome were recruited, who met the diagnosis of both “phlegm-dampness” and “internal-fire” according to ISTSFDS. ISTSFES was used to assess the syndrome severity; in our study it was used to assess the severity of phlegm-heat syndrome (phlegm-heat syndrome scores = phlegm-dampness syndrome scores + internal-fire syndrome scores).

## 1. Introduction

With the aging of the global population, stroke and related diseases have become a serious threat to human health [1]. Recent studies have shown that stroke has become the leading cause of death in China [2], in which ischemic stroke patients account for about 80% of all stroke patients [3] with a high morbidity. Traditional Chinese medicine (TCM) plays an important role in the prevention and treatment of strokes in China. The application of laboratory technology has been widely used for explaining the nature of the syndrome in stroke diseases. Moreover, biological markers have played an important role in the clinical diagnosis and treatment evaluation in many diseases. In general, biological markers closely associated with the pathogenesis of cerebral ischemia and reperfusion, blood-brain barrier damage, and cerebral infarction could be used for the diagnosis and clinical evaluation of acute ischemic stroke as a microindicator and are becoming a hot research topic.

Recent studies have demonstrated an association between stroke syndromes and changes in biological markers [4, 5]. For example, interleukin-6 (IL-6) and tumor necrosis factor-*α* (TNF-*α*) have been demonstrated to be positively associated with the fire-heat syndrome scores in acute ischemic stroke patients and it was suggested that elevated IL-6 and TNF-*α* levels can be used as indicators to distinguish between the fire-heat and non-fire-heat syndrome [6]. For a biomarker to be of optimal use, it should be rapid, cost-effective, specific, sensitive, as is the case for B-type natriuretic peptide (BNP) in the assessment of congestive heart failure [7]. However, there is still a lack of specific biomarkers for diagnosis of TCM syndromes of acute stroke [8].

Currently, the “integrating disease and syndrome” evaluation model is a widely acceptable model for clinical efficacy evaluation of TCM stroke by the TCM scholars [9]. This model combines the evaluation methods of Chinese and western medicine. For instance, at the disease level, the model adopted the US National Institutes of Health Stroke Scale (NIHSS) [10], Barthel Index (BI) [10], and Patient-Reported Outcome (PRO) [11], which are mature efficacy evaluation scales in western medicine. The evaluation of the level of the syndrome used the “Ischemic Stroke TCM Syndrome Factor Diagnostic Scale (ISTSFDS)” and “Ischemic Stroke TCM Syndrome Factor Evaluation Scale (ISTSFES)” [12]. Although the objectivity of clinical efficacy evaluation has been improved by the combination of these scales, the model lacks accurate and objective laboratory parameters to evaluate the clinical efficacy of acute stroke [13].

Analyzing current literatures linked with ischemic stroke, we found some novel biological indicators as follows: NDKA (nucleoside diphosphate kinase A) is an ubiquitous enzyme that catalyzes the exchange of terminal phosphate between different nucleoside diphosphates and is thought to be involved in the ischemic cascade after stroke [14, 15]. Previous studies found that NDKA was an early biomarker since its level was already elevated in blood of patients within 3 h after the stroke onset [15, 16]. The excitatory NMDA (N-methyl-D-aspartate) receptor is one of the key regulators of nerve cell membrane functions in the process embolic or thrombotic vascular occlusion stimulating the cascade of neurotoxicity, causing biochemical changes in brain tissue, the blood-brain barrier, and brain vessels. Recently, researches had investigated antibodies to the glutamate NDMA-R that had a 97% sensitivity and 98% specificity to distinguish ischemic stroke from control at 3 h [17]. PARK7 (Polyploidy-Associated Protein Kinase) is a highly conserved protein with unclear function. Allard et al. found that serum PARK7 levels were significantly increased in stroke patients 30 min to 3 hrs after onset with a sensitivity of 54%–91% and specificity of 80%–90% in diagnosis of stroke [15]. The studies found that UFDP (ubiquitin fusion degradation protein 1) was increased in human postmortem CSF, a model of global brain insult, and also was released in the blood of patients affected by a brain injury [18]. These biomarkers maybe used in clinical practice in further as indexes for diagnosis in acute ischemic stroke.

In this study, we examined the association between serum NDKA, NMDA, PARK7, and UFDP levels and the phlegm-heat syndromes of acute ischemic stroke and explored their value in evaluating the clinical efficacy of stroke.

## 2. Materials and Methods

### 2.1. Participants

Biological samples and medical records of fifty-one stroke patients with phlegm-heat syndrome that met the inclusion and exclusion criteria were obtained to form a biomarkers and clinical information database, which was established since January 2009 by the Clinical Research Centers of Dongzhimen Hospital, Beijing University of Chinese Medicine. The inclusive criteria were (1) meeting the diagnosis of cerebral infarction according to the “2010 China acute ischemic stroke diagnosis and treatment guidelines”; (2) both the diagnosis of “phlegm-dampness” and “internal-fire” existing (phlegm-dampness syndrome scores ≥10 points, internal-fire syndrome scores ≥10 points) according to the “Ischemic Stroke TCM Syndrome Factor Diagnostic Scale (ISTSFDS)”; (3) NIHSS scores being ≥5 points, but ≤22 points; (4) onset being within 72 hours; and (5) age being ≤75 years and ≥35 years. The exclusion criteria were (1) patients who were diagnosed with transient ischemic attack or cerebral hemorrhage or subarachnoid hemorrhage and (2) patients who had drag-over disease with liver, kidney, hematopoietic system, and endocrine system diseases, bone and joint diseases, mental illness, dementia, stroke sequelae, and stroke caused by brain tumor, traumatic brain injury, blood disease, and rheumatic heart disease. Ninety-five age and gender matched healthy people were recruited as controls. Signed informed consents were obtained from all subjects.

### 2.2. Clinical Evaluation

Basic information such as age and sex and fasting blood samples were collected from healthy controls. The clinical data for each patient was collected by the neurologist.

#### 2.2.1. General Information

The demographic data, medical history, lifestyle, blood test information (blood count, blood chemistry, blood clotting, etc.), and imaging data (CT or head MRI) of the 51 patients were collected at the day of recruiting.

#### 2.2.2. Biological Samples

Five mL of venous blood was collected from patients within 3 days of onset and 7 and 14 days after onset. The serum NDKA, NMDA, PARK7, and UFDP levels were measured using the enzyme-linked immunosorbent assay kits by following the manufacturer's instruction.

#### 2.2.3. The Observations of Therapeutic Efficacy

**Figure 1:**
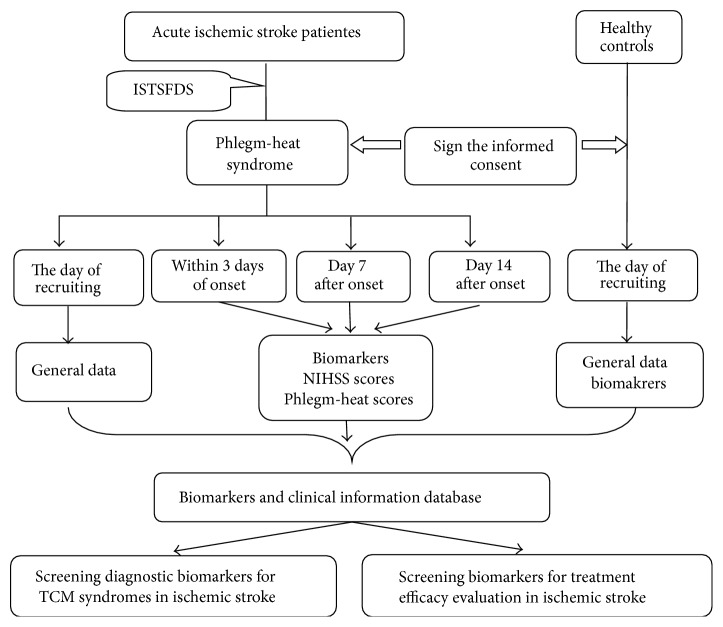
Flow chart of the study.

The evaluation of clinical therapeutic efficacy in stroke patients included disease evaluation and syndrome evaluation. Disease evaluation used the NIHSS (National Institutes of Health Stroke Scale) to assess the neurological impairment. Syndrome evaluation used the “Ischemic Stroke TCM Syndrome Factor Evaluation Scale (ISTSFES)” to assess the syndrome severity (phlegm-heat syndrome scores = phlegm-dampness syndrome scores + internal-fire syndrome scores). Evaluation point is within 3 days of onset and at day 7 and day 14 after onset (Figure 1).

### 2.3. Statistical Analysis

Statistical analyses were performed using SPSS 21 software (IBM, NC, USA). Data with normal distribution were expressed as mean ± standard errors. If the data showed homogeneity of variance, differences between groups were analyzed using independent samples *t*-test. If not, differences between groups was analyzed using nonparametric tests (Kruskal-Wallis *H* test). The receiver operating characteristic curve (ROC) analysis was used to elucidate the diagnostic value of biological markers for phlegm-heat syndrome. Single factor repeated measures analysis was used for assessment of the variance in biological indicators, NIHSS scores, and phlegm-heat syndrome scores in stroke patients within 3 days of onset and at 7 and 14 days after onset. Linear regression analysis was used for the correlation between changes of biological parameters and NIHSS scores or phlegm-heat syndrome scores. *P* < 0.05 was considered statistically significant.

## 3. Results

### 3.1. General Data

This study included 95 healthy controls and 51 patients. The general characteristics, including gender and age of healthy controls, were collected, and, beyond that, diseases of hypertension, diabetes mellitus and so forth of patients were collect (Table 1).

### 3.2. Serum NDKA, NMDA, PARK7, and UFDP Levels and Diagnosis Value

**Figure 2:**
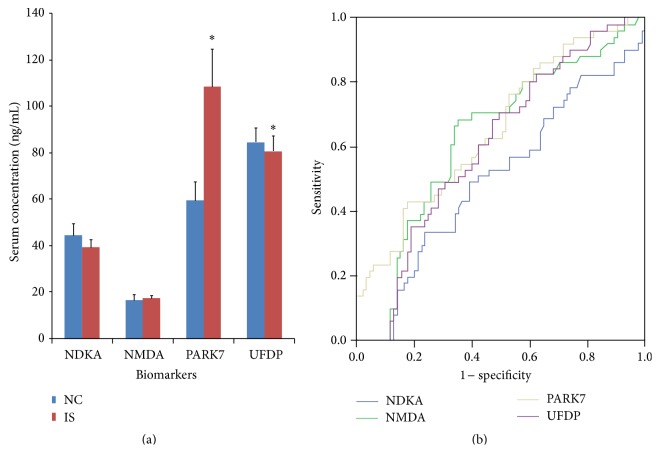
Serum NDKA, NMDA, PARK7, and UFDP levels and diagnostic value of phlegm-heat syndrome in acute ischemic stroke. (a) Comparison of serum NDKA, NMDA, PARK7, and UFDP levels between stroke patients within 3 days of onset and healthy controls. ^*∗*^ *P* < 0.05 versus controls. (b) Receiver operator characteristic (ROC) curves of serum NDKA, NMDA, PARK7, and UFDP levels. The area under ROC was 0.501, 0.639, 0.669, and 0.634, respectively.

Differences in these four biomarkers between ischemic stroke patients within 3 days of onset and healthy controls was analyzed using independent samples *t*-test or nonparametric tests (Kruskal-Wallis *H* test). The result showed a significant difference in serum PARK7 (*P* = 0.003) and UFDP (*P* = 0.045) between groups; in contrast, there was no significant difference in serum NDKA (*P* = 0.384) and NMDA (*P* = 0.774) between groups. Comparing the mean of these two groups, we found that the serum NMDA and PARK7 levels were higher than that in the healthy controls, whereas the serum NDKA and UFDP levels were lower than that in the healthy controls (Figure 2(a)).

Receiver operator characteristic curves (ROC) were used to evaluate the diagnostic value of the biological markers in the early stage of stroke with phlegm-heat syndrome. Results showed that NMDA, PARK7, and UFDP exhibited certain diagnostic accuracy with an AUC of 0.639, 0.669, and 0.634, respectively, and specificity of 54.1%–83.5%. The cut-off values of serum NMDA, PARK7, and UFDP levels for the diagnosis of phlegm-heat syndrome were 11.465 ng/mL, 74.152 ng/mL, and 46.950 ng/mL, respectively (Figure 2(b)). Serum NDKA level showed no diagnostic accuracy for the phlegm-heat syndrome (AUC 0.501) (Figure 2(b)).

### 3.3. Dynamic Changes in NDKA, NMDA, PARK7, and UFDP Levels

**Figure 3:**
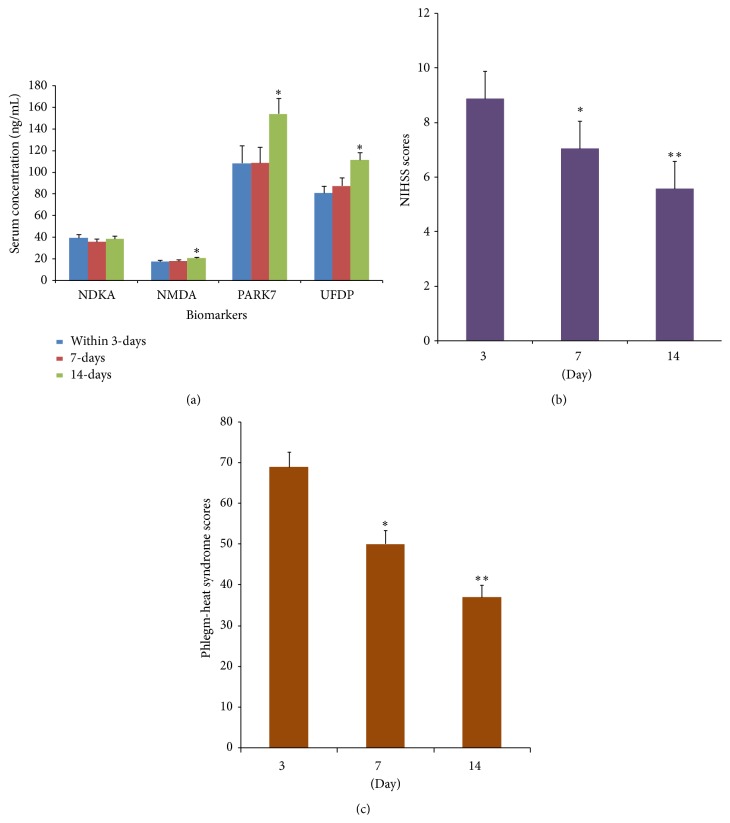
Dynamic changes of serum biological markers and scale scores of therapeutic efficacy in patients within 3 days of onset and 7 and 14 days after onset of stroke. (a) Serum NDKA, NMDA, PARK7, and UFDP levels in patients within 3 days of onset and 7 and 14 days after onset of stroke. ^*∗*^ *P* < 0.05 (14 days versus 7 days and 3 days). (b) NIHSS scores within the onset of 3 days and 7 and 14 days after onset. ^*∗*^ *P* < 0.05 (3 days versus 7 days), ^*∗∗*^ *P* < 0.05 (7 days versus 14 days). (c) Phlegm-heat syndrome scores within the onset of 3 days and 7 and 14 days after onset. ^*∗*^ *P* < 0.05 (3 days versus 7 days), ^*∗∗*^ *P* < 0.05 (7 days versus 14 days).

Single factor repeated measures analysis was used to measure the dynamic changes in serum NDKA, NMDA, PARK7, and UFDP levels within 3 days of onset and 7 and 14 days after the onset. Mauchly sphericity test showed that there was significant association between repeated measures data of NDKA (*W* = 0.841, *P* = 0.014) and PARK7 (*W* = 0.664, *P* = 0.000) and the need for freedom correction. In contrast, no significant associations between repeated measures data of NMDA (*W* = 0.936, *P* = 0.198) and UFDP (*W* = 0.954, *P* = 0.318) were observed and there was no need for freedom correction. ANOVA analysis showed a significant difference in serum NMDA (*F* = 5.301, *P* = 0.006), PARK7 (*F* = 17.472, *P* = 0.000), and UFDP (*F* = 10.518, *P* = 0.000) levels between 3 time points. In contrast, no significant difference in serum NDKA (*F* = 0.784, *P* = 0.446) level was observed between 3 time points. The pairwise comparison showed that the serum level of NMDA within 3 days was not significantly different from the level at 7 days (*P* = 0.564), whereas significant differences in serum NMDA levels were observed between the levels within 3 days and 14 days (*P* = 0.009) and between 7 days and 14 days (*P* = 0.009). Similarly, serum PARK7 and UFDP levels within 3 days were not significantly different from the levels on day 7 (*P* = 0.977 and *P* = 0.868, resp.), but significant differences were observed between the levels within 3 days and at day 14 (*P* = 0.000) and between day 7 and day 14 (*P* = 0.000 and *P* = 0.009, resp.) (Figure 3(a)). These observations suggested that serum NMDA, PARK7, and UFDP levels showed an increasing trend within 14 days of onset.

### 3.4. Correlations between Serum NDKA, NMDA, PARK7, and UFDP Levels and Treatment Efficacy Evaluation in Stroke Patients

#### 3.4.1. Correlations between These Biomarkers Levels and the Therapeutic Efficacy of Disease

The NIHSS Scale (NIH Stroke Scale) was used to evaluate the severity of defects in neural function in acute stroke patients as to the severity of stroke disease within 3 days of onset and at day 7 and day 14 after the onset of stroke. Single factor repeated measures analysis was used to measure its dynamic changes. Mauchly sphericity test revealed a significant association between repeated measures NIHSS scores (*W* = 0.621, *P* = 0.000) and the need for freedom correction. ANOVA analysis showed a significant difference in the NIHSS scores (*F* = 57.641, *P* = 0.000) between 3 time points. The pairwise comparison of two time points showed significant difference in NIHSS scores between the scores within 3 days and at day 7 or day 14 after onset (*P* = 0.000) and between day 7 and day 14 after onset (*P* = 0.000). These observations suggested that NIHSS scores were decreased gradually, indicating the gradual improvement in neurological functions and disease condition (Figure 3(b)).

Correlation analysis between changes of day 7 and day 14 serum NDKA, NMDA, PARK7, and UFDP levels (dependent variable) and NIHSS scores (independent variable) showed that serum NDKA, NMDA, PARK7, and UFDP levels were not significantly associated with NIHSS scores (*P* > 0.05) (Table 2). These results suggested that serum NDKA, NMDA, PARK7, and UFDP levels are not biomarkers to evaluate the therapeutic efficacy of disease in stroke patients.

#### 3.4.2. Correlations between These Biomarkers Levels and the Therapeutic Efficacy of Phlegm-Heat Syndrome

The ischemic stroke syndrome rating scale was used to evaluate the severity of syndrome within 3 days and at day 7 and day 14 of the onset of stroke. Single factor repeated measures analysis was used to measure its dynamic changes. Mauchly sphericity test revealed a significant association between repeated measured phlegm-heat syndrome scores (*W* = 0.655, *P* = 0.000) and the need for freedom correction. ANOVA analysis showed a significant difference in the phlegm-heat syndrome scores between the 3 time points (*F* = 62.462, *P* = 0.000). The pairwise comparison of two time points showed significant difference in phlegm-heat syndrome scores between the scores within 3 days and at day 7 or day 14 after onset (*P* = 0.000) and between day 7 and day 14 (*P* = 0.000). These observations suggested that phlegm-heat syndrome scores were decreased gradually, indicating the gradual improvement in phlegm-heat syndrome (Figure 3(c)).

Correlation analysis between changes of day 7 and day 14 serum NDKA, NMDA, PARK7, and UFDP levels (dependent variable) and phlegm-heat syndrome scores (independent variable) showed that serum NDKA, NMDA, PARK7, and UFDP levels were not significantly associated with the phlegm-heat syndrome scores (*P* > 0.05) (Table 3). These results suggested that serum NDKA, NMDA, PARK7, and UFDP levels are not biomarkers to evaluate the therapeutic efficacy of phlegm-heat syndrome in stroke patients.

## 4. Discussion

This study included 95 healthy subjects and 51 acute ischemic stroke patients with phlegm-heat syndrome. The serum NDKA, NMDA, PARK7, and UFDP levels; NIHSS scores; and phlegm-heat syndrome scores were measured within 3 days of onset and at 7 days and 14 days after the onset of stroke in 51 acute ischemic stroke patients. The associations between the serum levels of the tested biomarkers and phlegm-heat syndrome in acute ischemic stroke were analyzed within 3 days of the onset. The diagnostic value of each biological marker on the phlegm-heat syndrome in acute stroke patients was analyzed by ROC curve. The association between these biomarkers and treatment efficacy evaluation was analyzed by exploring the correlation of dynamic changes between the test biomarkers levels and the NIHSS scores as well as the phlegm-heat syndrome scores within 3 days of onset and at 7 days and 14 days after the onset. Our results demonstrated that the serum PARK7 and UFDP concentration have diagnostic value for the phlegm-heat syndrome of acute ischemic stroke. However, serum NDKA, NMDA, PARK7, and UFDP levels are not biomarkers for predicting the therapeutic efficacy of the disease and the phlegm-heat syndrome.

Previous studies have revealed that NDKA, NMDA, PARK7, and UFDP levels were significantly elevated in stroke patients within 3 h of onset and had certain sensitivity and specificity in the diagnosis of ischemic stroke [14–18]. This study found that serum PARK7 and UFDP levels in stroke patients with phlegm-heat syndrome within 3 days of onset were statistically different from the healthy subjects. The serum PARK7 level was elevated whereas UFDP level was lowered. The ROC curve analysis showed that serum PARK7 and UFDP levels had some diagnostic value on the phlegm-heat syndrome in ischemic stroke patients. However, currently, there were no reports on the associations between serum PARK7 and UFDP levels and other diseases such as coronary heart diseases and hypertension as well as stroke patients without phlegm-heat syndrome. Therefore, serum PARK7 and UFDP levels could not be used as a marker for the diagnosis of stroke with phlegm-heat syndrome currently. In addition, this study looked at the changing trends of serum NDKA, NMDA, PARK7, and UFDP levels and found that serum NDKA levels were not significantly changed within 3 days of onset to day 14 after onset. While no significant differences in the serum NMDA, PARK7, and UFDP levels were observed between the levels within 3 days of onset and at day 7 after onset, significant differences in serum levels of these 3 markers were observed between day 7 and day 14 after onset, and their levels had a tendency of increase. Our findings provided a complement to previous findings.

This study analyzed the associations between biological parameters and the clinical efficacy of acute stroke patients at the disease level and syndrome level. At the disease level, these biological markers showed no significant association with NIHSS scores at 3 time points. This indicated that these biomarkers lack value for evaluating the severity of defects in neural function in acute stroke patients. At the syndrome level, these biological markers exhibited no significant associations with the phlegm-heat syndrome scores and less efficacy in evaluating the severity of phlegm-heat syndrome. Therefore, these biomarkers cannot be used as indicators of clinical efficacy of therapy in acute stroke patients with phlegm-heat syndrome. However, this study found that the serum NMDA, PARK7, and UFDP levels had a tendency of increase at day 14 compared to day 7 after onset. Similarly, the NIHSS scores and phlegm-heat syndrome scores showed a tendency of decrease at day 14 compared to day 7 after onset. These observations may suggest a relationship between the biological markers and the clinical efficacy of therapy. No significant differences were observed, which may be a bias caused by the small sample size. Despite the failure to obtain statistical difference, using biological markers to evaluate the therapeutic efficacy of Chinese medical syndromes is a new idea.

We acknowledge the limitations in the present study. (1) This study lacks control of syndromes and cannot determine the relevance between these biological markers and other disease syndromes of stroke. (2) The sample size was relatively small and may cause biases in outcomes.

## 5. Conclusion

Serum PARK7 and UFDP levels within 3 days of onset of acute stroke with phlegm-heat syndrome have some diagnostic value in phlegm-heat syndrome. The serum NDKA, NDMA, PARK7, and UFDP levels did not show a significant tendency in day 7 compared to day 3 within onset, while the serum NDMA, PARK7, and UFDP, but not NDKA, levels exhibited a tendency of increase in day 14 compared to day 7. Although the tendency of increase of NDMA, PARK7, and UFDP levels shown parallels the decreases in NIHSS scores and phlegm-heat syndrome scores at day 14 compared to day 7 after onset, no statistical significance between the serum levels of these biomarkers and treatment efficacy evaluation was obtained. A study with larger sample size and a control of syndromes is necessary to establish the statistical association between these biological markers and the therapeutic efficacy of acute stroke.

## Figures and Tables

**Table 1:** General data.

Items	Patients	Healthy controls
*N*	51	95
Age (Yr., $\overset{-}{x}\pm s$)	62.27 ± 9.27	31.23 ± 9.74
Females (case (%))	43.1	46.3
Hypertension (case (%))	28 (54.9)	NA
Coronary heart disease (case (%))	9 (17.6)	NA
Diabetes mellitus (case (%))	9 (17.6)	NA
Hyperlipidemia (case (%))	16 (31.4)	NA
Previous stroke (case (%))	22 (43.1)	NA
Smoking (case (%))	18 (35.3)	NA
Alcohol (case (%))	10 (19.6)	NA

*Note*. NA indicates not applicable.

**Table 2:** Correlation between NIHSS scores and serum NDKA, NMDA, PARK7, and UFDP levels.

Dependent variables	Independent variable	*r*	*P*
NDKA	NIHSS scores	−0.056	0.696
NMDA		0.050	0.727
PARK7		0.086	0.546
UFDP		0.149	0.295

**Table 3:** Correlation between phlegm-heat syndrome scores and serum NDKA, NMDA, PARK7, and UFDP levels.

Dependent variables	Independent variable	*r*	*P*
NDKA	Phlegm-heat syndrome scores	0.074	0.608
NMDA		−0.206	0.146
PARK7		−0.111	0.437
UFDP		−0.218	0.125
